# The SIMI Gender ‘5 Ws’ Rule for the integration of sex and gender-related variables in clinical studies towards internal medicine equitable research

**DOI:** 10.1007/s11739-022-03049-y

**Published:** 2022-08-06

**Authors:** Valeria Raparelli, Francesca Santilli, Alberto Maria Marra, Giulio Francesco Romiti, Elena Succurro, Anna Licata, Elena Buzzetti, Salvatore Piano, Maristella Masala, Patrizia Suppressa, Cecilia Becattini, Maria Lorenza Muiesan, Giuseppina Russo, Chiara Cogliati, Marco Proietti, Stefania Basili, Italian Society of Internal Medicine (SIMI)

**Affiliations:** 1 Department of Translational Medicine, University of Ferrara, Via dei Borsari 46, 44121 Ferrara, Italy; 2 University Center for Studies on Gender Medicine, University of Ferrara, Ferrara, Italy; 3 Faculty of Nursing, University of Alberta, Edmonton, AB Canada; 4 Department of Medicine and Aging, and Center for Advanced Studies and Technology (CAST), University of Chieti, Chieti, Italy; 5 Department of Translational Medical Sciences, Italy and Center for Pulmonary Hypertension, Thoraxklinic, Member of the German Center for Lung Research (DZL), “Federico II” University of Naples, University Hospital Heidelberg, Heidelberg, Germany; 6 Department of Translational and Precision Medicine, Sapienza University of Rome, Rome, Italy; 7 Department of Medical and Surgical Sciences, University Magna Graecia of Catanzaro, Catanzaro, Italy; 8 Department of Health Promotion, Mother and Child Care, Internal Medicine and Medical Specialties (PROMISE), University of Palermo Medical School, Palermo, Italy; 9 Department of Medical and Surgical Science for Children and Adults, University of Modena and Reggio Emilia, Modena, Italy; 10 Unit of Internal Medicine and Hepatology (UIMH), Department of Medicine-DIMED, University and Hospital of Padua, Padua, Italy; 11 Department of Internal Medicine, University of Sassari, Sassari, Italy; 12 Department of Internal Medicine and Rare Disease Centre “C. Frugoni” University Hospital of Bari, Bari, Italy; 13 Internal and Cardiovascular Medicine—Stroke Unit, University of Perugia, Perugia, Italy; 14 Department of Clinical and Experimental Sciences, University of Brescia, Brescia, Italy; 15 Department of Clinical and Experimental Medicine, University of Messina, Messina, Italy; 16 Internal Medicine, Department of Biomedical and Clinical Sciences, L.Sacco Hospital, ASST Fatebenefratelli Sacco, University of Milano, Milan, Italy; 17 Department of Clinical Sciences and Community Health, University of Milan, Milan, Italy; 18 Geriatric Unit, IRCCS Istituti Clinici Scientifici Maugeri, Milan, Italy; 19 Liverpool Centre for Cardiovascular Science, University of Liverpool and Liverpool Heart & Chest Hospital, Liverpool, UK

**Keywords:** Sex, Gender, Clinical studies, Internal medicine, Data collection

## Abstract

Biological sex and sociocultural gender matter when it comes to health and diseases. They have been both proposed as the undeniable gateways towards a personalized approach in care delivery. The Gender Working Group of the Italian Society of Internal Medicine (SIMI) was funded in 2019 with the aim of promoting good practice in the integration of sex and gender domains in clinical studies. Starting from a narrative literature review and based on regular meetings which led to a shared virtual discussion during the national SIMI congress in 2021, the members of the WG provided a core operational framework to be applied by internal medicine (IM) specialists to understand and implement their daily activity as researchers and clinicians. The SIMI Gender ‘5 Ws’ Rule for clinical studies has been conceptualized as follows: Who (Clinical Internal Medicine Scientists and Practitioners), What (Gender-related Variables—Gender Core Dataset), Where (Clinical Studies/Translational Research), When (Every Time It Makes Sense) and Why (Explanatory Power of Gender and Opportunities). In particular, the gender core dataset was identified by the following domains (variables to collect accordingly): relations (marital status, social support, discrimination); roles (occupation, caregiver status, household responsibility, primary earner, household dimension); institutionalized gender (education level, personal income, living in rural vs urban areas); and gender identity (validated questionnaires on personality traits). The SIMI Gender ‘5 Ws’ Rule is a simple and easy conceptual framework that will guide IM for the design and analysis of clinical studies.

## Introduction

The awareness that biological sex (i.e., sex assigned at birth) and sociocultural gender (i.e., sociocultural norms roles and expectations) are relevant modifiers of health and disease has been a recent achievement by the life sciences researchers and clinicians [1]. Despite the advocacy of international societies and gender champions to promote the knowledge on sex and gender analysis for improving the quality of science, the terms are still often used interchangeably, yet they capture different aspects of a person. Specifically, gender refers to the psychosocial aspects of being a woman or a man (“psychosocial sex”) as opposed to the biological aspects of being male or female (“biological sex”) [2]. However, some males and females may report gender-related characteristics traditionally attributed to the opposite sex, and some individuals may identify as neither male nor female thereby leading to the increasing recognition of gender as a spectrum (rather than binary entity) in social sciences and the general public. As such, the distribution of gender-related characteristics within populations of men and women is likely to influence health differently than biological sex. To underline the different meaning of these terms, from 2012 the Canadian Institute of Gender and Health acknowledges that *“Every cell is sexed, and every person is gendered”* [3] pointing out how biological factors (sex-based) and psycho-socio-cultural factors (gender-related) contribute profoundly to shape who we are either in maintaining health status or in developing diseases. Furthermore, the concept of intersectionality between sex, gender, and other social factors (e.g., race, immigration status, etc.) is currently emerging to reflect the importance of diversity in health [4, 5]. An intersectional framework assumes that an individual’s experiences are not simply equal to the sum of their parts but represent intersections of social power’s axes. For example, the health-related experiences of immigrant women may be different from those of immigrant men and non-immigrant women. The term intersectionality, originally coined in the critical race theory is considered an extension of sex and gender analysis and can be applied across other social identities or positions in society [4, 5].

The specialty of internal medicine (IM) is an all-embracing medical discipline, dealing with all aspects of pathology and organ-based specialties [6]. The IM specialists face every day the challenges of a 360-degree approach to the care of complex and multimorbid and often older individuals. IM healthcare professional are those that would mostly benefit from a sex- and gender-based evidence as they traditionally build their clinical decisions on a multidimensional evaluation of patients beyond the disease-centered focus. Therefore, clinical investigators working in the IM field should recognize the value of a sex- and gender-oriented approach to answer relevant research questions and should be supported with strategies for incorporating sex and gender-related variables when conducting either pre-clinical or clinical studies.

The aim of this study was to build an operational framework that can guide IM researchers in implementing their approach giving the context and the content on how to practically perform it.

## Methods

During the 2019 National Congress of the Italian Society of Internal Medicine (SIMI) held in Rome (Italy), a Working Group (WG) on Gender Medicine has been implemented with the specific aim of promoting the integration and application of sex- and gender-oriented approaches in internal medicine (IM) from research to clinical practice. In this context, the first goal of the WG was to implement the strategy for the collection of gender-related variables in the no-profit observational studies promoted by SIMI. Starting from a narrative detailed and extensive literature analysis on the applicable tools for the gender-based data acquisition in human studies, the WG met on regular basis and discussed the most simple and feasible strategy for promoting among the IM community. In a dedicated session of the virtual 2021 SIMI National Congress, the WG collegially agreed on the most appropriate approach for the implementation of a sex- and gender-sensitive framework in clinical studies promoted by the SIMI. Specifically, the WG SIMI Gender has formulated a short three-question questionnaire to assess whether the session participants were able to define properly the terms ‘sex’ and ‘gender’. Then the core dataset of variables to be incorporated in the design of the clinical studies promoted by SIMI was voted, and afterwards the perception of participants regarding the best way to inquire patients on their gender identity was discussed.

## Results

**Fig. 1:**
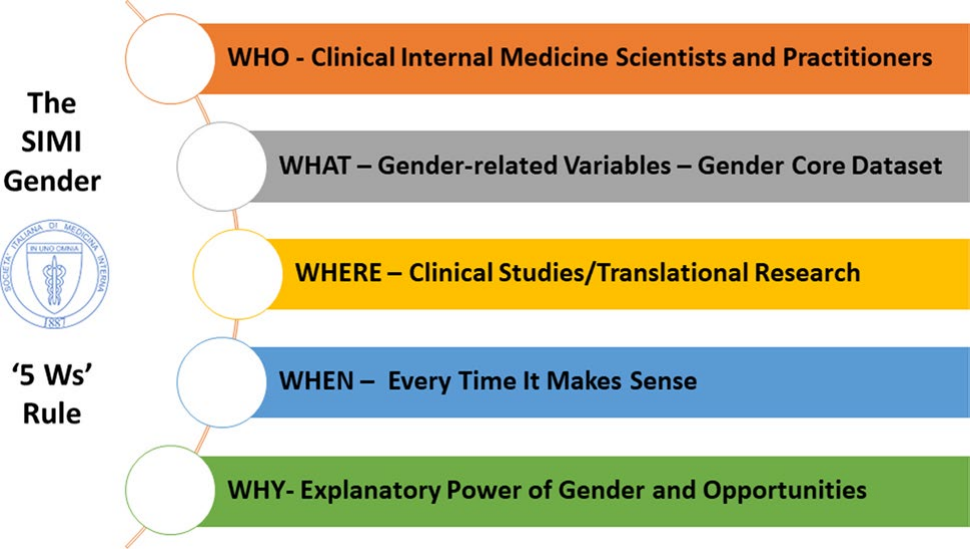
The SIMI Gender ‘5 Ws’ Rule for clinical studies

The activities of the WG SIMI Gender led to the operational definition of the SIMI Gender ‘5 Ws’ Rule for clinical studies which have been conceptualized as summarized in *Who, What, Where, When, and Why* (Fig. 1).

### Who—clinical internal medicine scientists and practitioners

In their clinical activity IM specialist often rely practice on evidence that do not always reflect the real-world scenarios [6]. For example, in drugs prescription while it is well-recognized that the proportion of female participants to interventional randomized control trials is far lower than the male counterpart, the results on drugs efficacy and safety are usually transferred and generalized to all people, when they should not [7]. According to data arising from a survey started by the ‘Internal Medicine Assessment of Gender differences IN Europe’ (IMAGINE), a working group of the European Federation of Internal Medicine, the largest society of Internists in Europe, the awareness of sex/gender issues in the European internal medicine community is already high, reaching approximately 80% of participants [8]. Therefore, the internal medicine community represents a solid bedrock where implement sex/gender strategies when it comes to planning research activities and clinical trials.

### What (and how) gender-related variables—gender core dataset

The collection of gender-related variables has been proposed in 20 s’ through questionnaires that included questions pertinent to each of the four domains that gender encompasses (i.e., gender identity, relations, roles, and institutionalized gender) [2]. Interestingly in 2016 Canadian investigators proposed the collection of gender-relevant variables through self-administered questionnaire and then developed a methodology to combine the data to generate a composite measure of gender, the gender score [9] as part of the ‘GENdEr and Sex DetermInantS of Cardiovascular Disease: From Bench to Beyond Premature Acute Coronary Syndrome’ (GENESIS-PRAXY) study [10]. Numerous variables (for the most part previously validated questions from literature), covering the four gender aspects (i.e., gender roles, gender identity, gender relations, and institutionalized gender) were identified and them to create a questionnaire for a cohort of young individuals with acute coronary syndrome. Through Principal Component Analysis (PCA), the Authors identified the gendered variables most associated with sex (primary earner status, personal income, number of hours per week doing housework, level of stress at home, Bem sex role inventory (BSRI) masculinity score and BSRI femininity score) and used them to create a composite gender score. Furthermore, gender score predicted recurrence of cardiovascular events, and showed that individuals with characteristics that society traditionally ascribed to women, regardless of sex, had worse outcomes. Some criticisms have been raised by such approach in clinical practice. One challenge in applying these methods is attributed to the use of a long questionnaire comprised of 32 components. This length renders it unpractical and not user-friendly among researchers. Moreover, designing a composite gender score may compromise the ability to identify which aspects of gender most importantly impact health outcomes. Finally, it is important to determine whether the results obtained from the GENESIS-PRAXY study, a cohort of patients with cardiovascular disease, are repeatable or different in other clinical contexts.

The applicability of the GENESIS-PRAXY methodology using retrospective cohorts have been tested in other contexts [11, 12] and systematically incorporated as a potential tool in the application of the ‘Gender Outcomes International Group: to Further Well-being Development’ (GOING-FWD framework [13–19]. The GOING-FWD methodology has led to understand more clearly the effect of gender on outcomes also in population-based health surveys and in diverse clinical scenarios. The most intriguing finding is that the gender-related factors included in the gender score measure vary by countries underlying the cultural and country-specific nature of gender [15–19].

As the debate on the best way for measuring the impact of gender on health outcomes is a moving field, other proposals have been published. In 2021, a gender assessment tool—the Stanford Gender-Related Variables for Health Research—for use in clinical and population research [20] has been proposed. Specifically, seven main variables should be captured in health research including caregiver strain, work strain, independence, risk-taking, emotional intelligence, social support, and discrimination.

Based on the abovementioned literature we have constructed an Italian version of the self-administered questionnaire to collect sex and gender-relevant variables in clinical studies (Table 1). The main findings of this interactive web-based discussion session were: (1) the majority of attendees were able to identify a gender-related variable; (2) the variables to be included in the core dataset should be gender relations (i.e., marital status, social support, discrimination); gender roles (i.e., occupation, caregiver status, household responsibility, primary earner, household dimension); and institutionalized gender (i.e., education level, personal income, living in rural vs urban areas); (3) validated questionnaires on personality traits are considered the most feasible approach to explore individuals’ perception of gender identity rather than a direct question *“how do you perceive your identity regardless of the sex you were assigned at birth”*.

**Table 1:** Examples of questions for the integration of gender domains in clinical studies

GENESIS-PRAXY Questionnaire (J Am Coll Cardiol. 2016)		
	Questions	Answers
Education	What is the highest level of education that you completed?	No degree, certificate or diploma Completed high school Some college/university Completed post-secondary school (college/university) Completed registered apprenticeship/or other trades certificate
Occupation	Which statements describe your current work situation?	Currently working Student Homemaker Unpaid volunteer Unemployed, looking for work On leave of absence Other (specify):
Primary earner	Are you the primary earner in your house?	Yes No
Housework load	On average, how many hours a week do you usually spend doing housework (e.g., cleaning, cooking, washing, etc.)?______________	0–168
Housework responsibility	Are you the primary person responsible for doing housework in your home?	Yes No
Stress perception	How do you rate the following? Stress level at work—I do not work Stress level at home Overall stress	Ten-point scale (No stress = 1, 10 = Most stress)
Personal income	What range is your personal income?	Less than $15,000 $15,000 to $29,999 $30,000 to $49,999 $50,000 to $69,999 $70,000 to $99,999 More than $100,000 Do not know Do not wish to answer
Caregiving role	Are you directly responsible for caring for children or other people living with you?	Yes No
Identity	Are you?	Man, woman, cis-gender, transgender, other, any
Mental burden	Have you ever received a diagnosis of anxiety disorder, depression or have you ever taken anxiolytics or anti depressive medications?	Anxiety (or anxiolytics) Depression (or anti depressive drugs) Both Never

Stanford Gender-Related Variables for Health Research (Biol Sex Differ 2021)		
	Questions	Answers
Caregiver strain	In the past year, How often did you feel physically exhausted because of your caretaking responsibilities? How often did you feel emotionally exhausted because of your caretaking responsibilities? How often have your caretaking responsibilities caused you to worry about the future? On average, how many hours per weekday do you spend on taking care of someone in need (caring for children, elders, partners in need, etc.)?	Five-point scale (Never = 1, 5 = Always) 0–24
Work strain	How often, does your job… Require working fast? Involve repetitive tasks? How often do you feel … Emotionally exhausted from your work activities? Physically exhausted from your work activities? On average, how many hours per weekday do you spend on the following: Work (paid work, studying, internships, etc.)?	Five-point scale (Never = 1, 5 = Always) 0–24
Independence	How important is it for you… To solve your problems on your own? To be independent?	Five-point scale (Not at all important = 1, Extremely important = 5)
Risk-taking	In general, how prepared are you to take risks? How prepared are you to take risks… When making financial decisions? When it comes to recreational activities?	Five-point scale (Not at all prepared = 1, Completely prepared = 5)
Emotional intelligence	How often … Do friends talk to you about their problems? Do you talk to your friends about your problems? How easy is it for you to express what you are feeling to others?	Five-point scale (Never = 1, 5 = Always) Five-point scale (Not at all easy = 1, Extremely easy = 5)
Social support	In the past year, how often did you have someone… To show you love and affection? To help you with daily chores?	Five-point scale (Never = 1, 5 = Always)
Discrimination	Because of your gender, how often have you felt discriminated… Against? Against when getting hired? Against when at school? Against when receiving medical care? Against in public settings? Against in your family?	Five-point scale (Never = 1, 5 = Always)

### Where—clinical studies/translational research

The members of the WG agreed that the impact of sex as biological variable and gender as sociocultural variable should be assessed in observational and cohort studies. Whenever there is a database with already collected clinical data or in designing a study to answer a specific research question, the availability of gender-related variables should be pursued.

Furthermore, since IM scientists may well be involved also in translational research, the members of the WG recognize that the integration of sex and gender-based approach should permeate every phase of research from bench to bedside. Identifying the sex and gender differences in the mechanism, disease or treatment under investigation is always a priority.

### When—Every time it makes sense

The member of the WG strongly recommend defining clearly from the beginning the gender-oriented conceptual framework that the clinical scientists expect to be plausible in a specific clinical context. In this light it is crucial to run preliminarily a literature review on what is already known in terms of sex and gender impacts on the exposures and outcomes of interest. The advice to clinical scientists is first to ask themselves “*Is gender as socio-cultural factor relevant for my research question?*”. Here both ‘yes’ and ‘no’ answers need to have a proper justification. Then, if the answer is yes, how the gender domains can be integrated into the research proposal (*i.e.,* research design, methods, analysis, interpretation, and dissemination of findings) should be addressed.

### Why—Explanatory power of gender and opportunities

The members of the WG defined the meaningfulness of the approach proposed. The main reason for integrating sex and gender domains in clinical research is to produce better, equitable and high-quality science. [21]

A sex- and gender-blinded approach in data collection can lead to false findings and increase the likelihood of missing critical opportunities to discover differences in the interplay between exposures and outcomes for individuals which could inform future interventions. When research fails to account for sex and gender there is a risk of harm in assuming that the results apply to everyone. Indeed, the explanatory power of sex and gender in overt differences of disease under study as well as a different response to treatment should favor the development of more tailored approaches to care. The understanding of how gender-related factors can generate differences in health outcomes across the spectrum of genders is undoubtedly a strategy to address any underlying causes of inequities.

## Discussion

The SIMI Gender ‘5 Ws’ Rule represents an easy and pragmatic guide for any internist interested in improving scientific research and disseminating scientific knowledge for the benefit of all patients. In the era of precision medicine, we are currently going through, sex and gender are the gateways to achieve a personalized approach in both research and clinical practice.

The bottom line of the Gender Medicine is that sex and gender permeate the life of people, therefore, when it comes to health they are theoretically always involved. Therefore, a sex and gender-based consideration should be pursued almost always, specifically when an impact on outcomes is envisaged. In fact, gender domains capture a huge group of factors that are broadly recognized as mediators or modifiers of outcomes. The collection of gender-related variables is still an unmet need and the truly gamebreaker when it comes to provide high-quality science. The *What* of the SIMI Gender ‘5 Ws’ Rule was built upon the previous literature exploring the feasibility of measure gender. In fact, the first hurdle clinician faces in the integration of sex and gender domains is to operationalize the definition of a gender-related variable. Specifically, a gender-related variable is a non-biological variable which differs in terms of magnitude, prevalence, and/or impact between people regardless of the sex they were assigned at birth. As shown by our literature review, there is a lack of a standardized method for embracing the multidimensionality of gender. Depending on the availability of data it might be worthy to test the effect of gender using a composite measure as was performed by GENESIS-PRAXY Investigators [9, 10]. The methodology of constructing the gender score has been validated since the inception in cohorts from retrospective and prospective studies [11–19]. Furthermore, international scientists have been supporting the assessment of even one domain or few individual gender-related factors depending on the availability of data [20–22].

The WG also acknowledges that over the last decade both funding agencies and medical journals are promoting for the excellence of science to assess a research protocol or publication based on the integration or omission of sex and/or gender. In fact, Canadian, American, and European research funding agencies supported by governments have implemented different strategies to favor the inclusion of sex and gender-based considerations in the grant’s procedure with the ultimate goal of awarding those protocols that integrate diversity and justify why sex and gender have been evaluated. [23]

Sex and gender matter when it comes to data analysis and reporting the findings of the clinical research. Of interest, stop controlling for sex [24] and favor sex-stratified analyses and interaction analyses [13, 14, 21] are recommendations that the scientific community is promoting to ensure equitable evidence at glance of practitioners for male and female individuals.

Finally, the International Committee of Medical Journal Editors recommends that the use of terms sex (when reporting biological factors) and gender (identity, psychosocial or cultural factors) should be guaranteed throughout the entire manuscript and, unless inappropriate, Authors should report the sex and/or gender of study participants, the sex of animals or cells, and describe the methods used to determine sex and gender [25].

## Conclusions

The IM community recognize that biological sex and sociocultural gender (i.e., societal norms, roles, and expectations) are relevant modifiers of health and disease. The application of sex and gender lenses in informing our understanding of health maintenance and disease development is key to personalize approaches and provide patient-centered care. The first obstacle to the integration of sex and gender in clinical practice and research is the lack of the habit to collect a granularity of sex- and gender-related variables in the case report forms of clinical research studies. The Gender WG of the SIMI identified and proposed an operational framework the SIMI Gender ‘5 Ws’ Rule to guide IM researchers in the challenging attempt of performing an equitable research with meaningful findings for improving the health of all patients.
